# Effects of Land Use, Topography and Socio-Economic Factors on River Water Quality in a Mountainous Watershed with Intensive Agricultural Production in East China

**DOI:** 10.1371/journal.pone.0102714

**Published:** 2014-08-04

**Authors:** Jiabo Chen, Jun Lu

**Affiliations:** 1 Department of Natural Resources, College of Environment and Natural Resources, Zhejiang University, Hangzhou, Zhejiang Province, China; 2 China Ministry of Education Key Lab of Environment Remediation and Ecological Health, Zhejiang University, Hangzhou, Zhejiang Province, China; Ecole des Mines d'Alès, France

## Abstract

Understanding the primary effects of anthropogenic activities and natural factors on river water quality is important in the study and efficient management of water resources. In this study, analysis of Variance (ANOVA), Principal component analysis (PCA), Pearson correlations, Multiple regression analysis (MRA) and Redundancy analysis (RDA) were applied as an integrated approach in a GIS environment to explore the temporal and spatial variations in river water quality and to estimate the influence of watershed land use, topography and socio-economic factors on river water quality based on 3 years of water quality monitoring data for the Cao-E River system. The statistical analysis revealed that TN, pH and temperature were generally higher in the rainy season, whereas BOD_5_, DO and turbidity were higher in the dry season. Spatial variations in river water quality were related to numerous anthropogenic and natural factors. Urban land use was found to be the most important explanatory variable for BOD_5_, COD_Mn_, TN, DN, NH_4_^+^-N, NO_3_^−^-N, DO, pH and TP. The animal husbandry output per capita was an important predictor of TP and turbidity, and the gross domestic product per capita largely determined spatial variations in EC. The remaining unexplained variance was related to other factors, such as topography. Our results suggested that pollution control of animal waste discharge in rural settlements, agricultural runoff in cropland, industrial production pollution and domestic pollution in urban and industrial areas were important within the Cao-E River basin. Moreover, the percentage of the total overall river water quality variance explained by an individual variable and/or all environmental variables (according to RDA) can assist in quantitatively identifying the primary factors that control pollution at the watershed scale.

## Introduction

The deterioration of river water quality has become a primary environmental concern due to unsustainable anthropogenic activities; the demand for freshwater has been rapidly increasing in many developing countries, especially in China [1]–[3]. River water quality is controlled by complex anthropogenic activities and natural factors at both the river and watershed scales [1], [7]–[9]. Understanding the temporal and spatial variations in river water quality and estimating the primary regional factors that affect water quality can assist researchers in establishing priorities for sustainable water management [2], [10]–[12]. The relationships between water quality parameters and land use/cover, population density and point source discharge have been frequently studied [1], [7], [9], [13]–[15]. Simultaneously, topography and animal waste discharge are considered important factors that affect watershed river water quality [7], [13]–[16]. Particularly, several countries, e.g., China, contain large mountainous areas [17] and extensive animal production that lack strict management techniques [16], [18], [19].

Recently, Pearson correlations have been widely employed to determine the relationships between environmental variables and river water quality [1], [7]. This method is simple and provides quantitative information; however, Pearson correlations lack visualization. Multiple regression analysis (MRA) is a useful tool that is commonly used to determine that watershed characteristics that best explain the spatial variability of an individual river water quality variable [7], [20]. This method lacks general information regarding pollution types. Moreover, principal component analysis (PCA) has become a widely accepted method in river water quality assessment and source apportionment studies in the last decade [2]. This method is commonly used to obtain the types of specific pollution sources without explanatory variables; however, PCA can only provide preliminary information regarding pollution types. Redundancy analysis (RDA), a multivariate statistical analysis method, has been proved useful for qualitative analysis of the interactions between river water quality and watershed characteristics in highly complex systems [13], [21], [22]. RDA can reveal the influences of environmental factors on overall water quality, not only on single water quality variables [20]. Several commonly used statistical methods for pollution source identification are complementary (i.e., they have their own benefits and limits). Summary of commonly used statistical methods on pollution source identification in recent years is listed in **Table S1**. However, comprehensive applications of different statistical methods to evaluate the effects of environmental variables on water quality have not been fully explored in river studies in China. Furthermore, few studies have used RDA analysis to quantitatively evaluate the effects of watershed characteristics on the overall river water quality.

The Cao-E River is located upstream of the Qiantang Estuary, which is a major riverine system in Zhejiang Province, China, and is the primary source of industrial, agricultural and domestic water supplies [10]. Furthermore, the blue algae bloom that occurred in July 2004 occurred in the Qiantang Estuary [23], [24].

In this study, ANOVA, Pearson correlation analysis, MRA, PCA and RDA were applied to investigate the effects of sub-watershed land use, topography and socio–economic factors (including animal waste discharge) on river water quality at the watershed scale for the Cao-E River system in a GIS environment. The objectives of this study were threefold: (1) to examine the temporal and spatial variations in water contamination within the study area, (2) to investigate the relationships between the river water quality parameters and land use, topography and socio-economic factors, and (3) to identify the primary pollution sources to estimate the possible sources that affect the water quality parameters in the Cao-E River basin. The results can be helpful for water conservation in the Cao-E River basin, and they provide a valuable tool for water quality agencies to develop assessment strategies for effective water quality management and rapid solutions for water pollution problems at the watershed scale.

## Materials and Methods

### 1 Ethics statement

No specific permits were required for the described field studies; the sampling did not cause any disturbance to the environment or to the protected species at the sampling sites.

### 2 Study area

**Figure 1:**
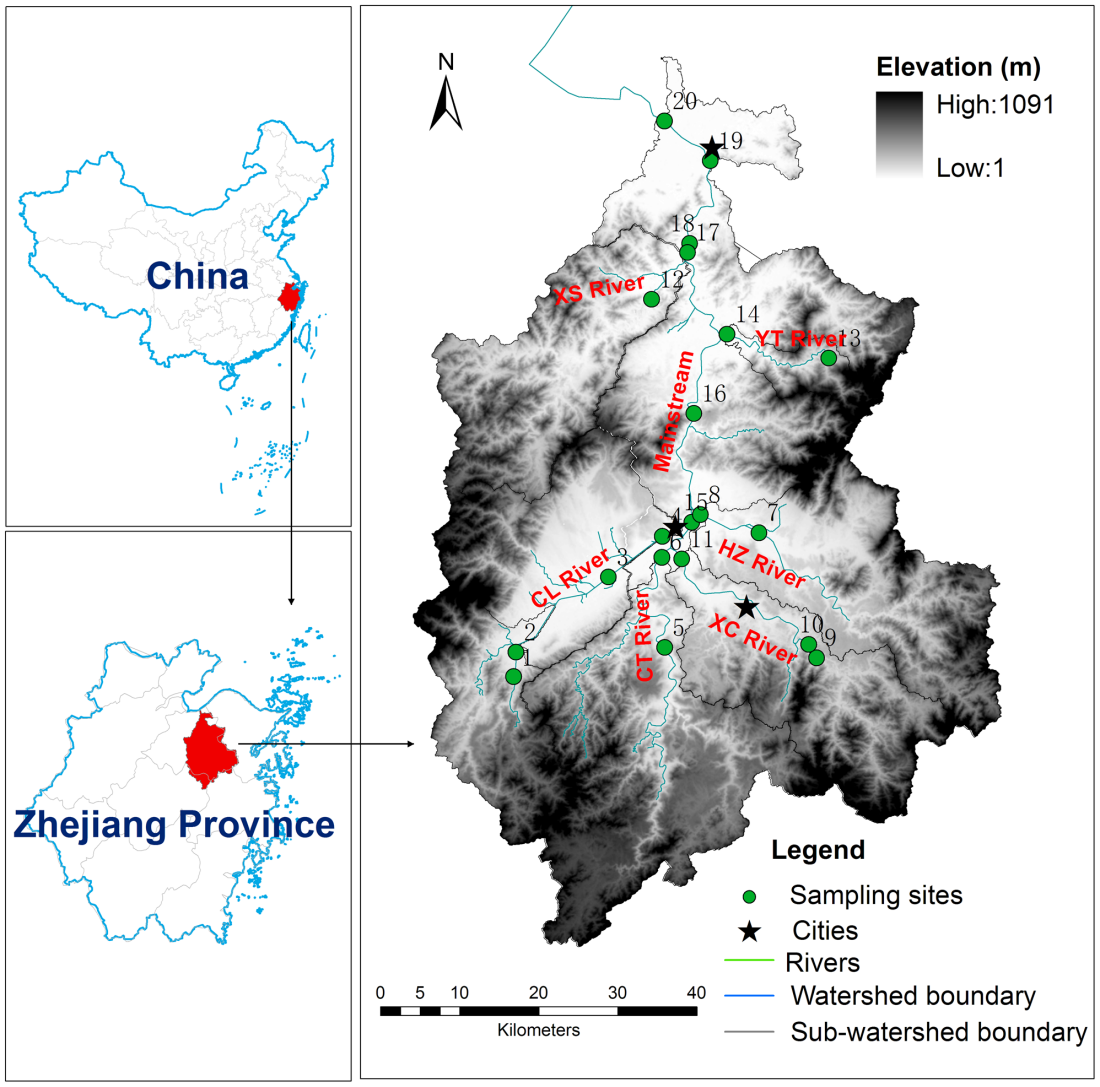
The Geographical Location Of The Sampling Sites In The Cao-E River System. (Sampling Stations 1-Cl1, 2- Cl2, 3-Cl3, 4-Cl4, 5- Ct 1,6-Ct2,7- Hz1,8- Hz2,9- Xc1,10- Xc2,11- Xc3,12- Xs1,13- Yt1,14- Yt2,15- M1,16- M2,17- M3,18- M4,19- M5, 20- M6).

The Cao-E River (29°08′-30°15′ N, 120°30′-121°15′ E) is located in Zhejiang Province, East China. The Cao-E River is one of the main tributaries of the Qiantang Estuary, which flows into the East China Sea. The river system contains a main stream and several major tributaries (i.e., the CT River, the XC River, the CL River, the HZ River, the XS River and the YT River; **Figure 1**). The main stream is ∼197 km long with an average slope of 3.0‰. The Cao-E River basin has a typical subtropical monsoon climate with an average annual rainfall of 1500 mm and an average ambient temperature of 16.2°C [25]. The watershed is within a mountainous region. Specifically, mountains and hills cover 2/3 of the area; the remaining area is covered by plains with intensive agricultural production [26].

### 3 Data Sources

#### Water quality data

The stream water samples (the number of samples used for statistical analysis is 36) were collected from July 2003 to June 2006 at 20 sampling sites throughout the watershed (Figure 1). Specific sampling stations that were largely influenced by point source pollution were excluded from further analysis. These specific sampling sites should meet the following conditions: (1) there was an obvious outlet of point pollution discharge near the sampling site, and (2) The standard deviation of the water quality parameter (BOD_5_) at the specific sampling sites were triple more than the standard deviation for total sampling sites. The samples were collected once a month between 9:00 am and 16:00 pm. Moreover, the water samples were collected at approximately 0.3 m below the water surface in the central stream, placed into plastic bottles (2.5 L), transported to the laboratory and stored at 0∼4°C for subsequent chemical analysis. The chemical measurements were performed in the laboratory within 24 h after collecting the water samples. Moreover, dissolved oxygen (DO), water temperature (T), pH and electrical conductivity (EC) were measured when the water samples were collected with a hand-held multi-parameter instrument (Multi 340i SETs, The Merck Co. Ltd., Germany). The turbidity was measured with a hand-held Turbiquant 1000IR (Multi 340i, The Merck Co. Ltd., Germany). The following chemical and biological water quality parameters were measured: chemical oxygen demand (COD_Mn_), 5-day biochemical oxygen demand (BOD_5_), total nitrogen (TN), total phosphorus (TP) and dissolved phosphorus (DP), which were measured using standard methods [27]. The ammonium nitrogen (NH_4_^+^-N) content was measured using an Astoria analyzer system (AAS, Brown Rupee CO. Ltd., Germany) after filtration through a 0.45-µm filter (Hailing Medicine, Zhejiang Province, China). The nitrate nitrogen (NO_3_^−^-N) content was measured from the absorbance of the sample at 220 nm (A220) and 275 nm (A275) using a visible and ultraviolet spectrophotometer (Shanghai Spectrum Instruments, Shanghai, China) after filtration through a 0.45-µm filter; the results were determined by the following relationship: A220 - 2×A275 [27].

#### Land use/cover, topography and socio-economic data and GIS analysis

Latitude and longitude were measured using a hand-held GPS. The catchment boundaries were delineated on a 30-m spatial resolution digital elevation model (DEM); the stream network was represented using the Soil and Water Assessment Tool (SWAT) model. ArcGIS 9.3 Desktop GIS software was used to calculate the watershed characteristics for each sampling site. All datasets were converted to a common digital format (WGS 1984) and a common coordinate system (Albers Equal Area). Hydrologic units were not solely defined by the watershed above an individual point [28]; a hydrologic unit on the Cao-E River may cover 2/3 or more of the entire watershed above that sampling station (most hydrologic units on tributaries encompass the entire watershed above a sampling station).

The socioeconomic data, including land use change, the gross domestic product, animal husbandry output and human population in the watershed, were obtained from the Shengzhou (SZ), Xinchang (XC), Shangyu (SY) and Shaoxing (SX) County Statistical Yearbooks for the period 2003–2006 and were adjusted with some typical investigation in several towns within the watershed. The temporal variations in land use were considered to be minimal. The annual changes in forest areas and water areas were less than 0.05%, the annual decrease rate in cropland areas was less than 1% and the annual increase rate in urban areas was less than 2.5%., respectively, for the entire watershed and throughout the study period. Thus, Landsat Thematic Mapper imagery from 2006 was used to map the land cover in the study area [29]. The land cover was categorized into the following 4 classes: forest, water, cropland and urban land. The annual increases in human population density, gross domestic product per capita and animal husbandry output per capita were less than 1.5%, 10% and 2%, respectively, for the entire watershed and throughout the study period. Therefore, the averages of the socio-economic data for the entire study period were selected in this study. The slope map was derived from the DEM using a GIS spatial analyst tool (ESRI, 2006). The land use, slope, DEM and administrative map (with attribute data, including the gross domestic product, animal husbandry output and human population for every town) were used to calculate the respective land use area (forest, water, urban land and cropland), mean elevation, mean slope, area, human population density, gross domestic product per capita and animal husbandry output per capita within each sub-watershed using a GIS spatial analyst tool. The abbreviations and statistics of the sub-watershed characteristics in the Cao-E River basin are summarized in **Table 1**.

**Table 1:** Abbreviations And Basic Statistical Information Of The Sub-Watershed Characteristics In The Cao-E River Basin.

	Parameters	Abbreviation	Range	Mean ± Sd
Land Use	Forest Percentage (%)	Frt	25.72–74.50	50.31±12.36
	Water Percentage (%)	Wt	0.13–3.39	1.87±0.87
	Urban Percentage (%)	Ub	0.45–14.44	4.09±3.52
	Cropland Percentage (%)	Crp	21.58–57.73	43.85±10.18
Topography	Mean Elevation (M)	Elv	125.12–556.54	306.17±116.17
	Mean Catchment Slope (°)	Slp_mean_	10.20–17.72	13.74±2.29
	Catchment Area (Km^2^)	Ar	38.39–2286.21	764.53±674.58
Socio-Economic Factors	Human Population Density (Persons/Km^2^)	Hp_d_	123.01–576.68	316.11±119.12
	Gross Domestic Product Per Capita (10^5^ Chinese Yuan)	Gdp_pc_	135.51–2151.16	582.38±427.88
	Animal Husbandry Output Per Capita (10^5^ Chinese Yuan)	Aho_pc_	8.07–39.40	20.56±8.61

### 4 Data analysis

Analysis of variance (ANOVA) was used to compare river water quality variations between different seasons (i.e., rainy and dry) via the Student-Newman-Keuls multiple-range test (P<0.05). Relationships among the considered variables were tested using Pearson's coefficient with statistical significance set at P<0.05. Major gradients and principal patterns in the water quality data between wet and dry seasons were detected using principal component analysis (PCA). PCA transforms a dataset consisting of *p* variables (analytical constituents) that are interrelated or correlated to various degrees into a new dataset containing *p* new orthogonal and uncorrelated variables; these variables are called the principal components (PCs) [30], [35]. The PCs are linear functions of the original variables in which the sum of their variances is equal to that of the original variables [30]. The PCs following a descending ordered according to their variances with PC1 corresponding to the variable with the largest variance. Algebraically, for *p* original variables (i.e., *x*_1_, *x*_2_,…, *x*_p_). Additional PCs up to *p* can be formulated in a similar manner. The variances of the PCs are the eigenvalues; the coefficients or weights, are the eigenvectors extracted from the covariance or correlation matrix. In the example PCA, the correlation matrix was used in all cases. The goal of PCA is that the first *k* PCs (where *k*<<*p*) retain most of the information in all of the *p* original variables, which effectively reduces the practical dimensionality of the dataset. More specifically, if the correlations are high among many of the original variables, the first few PCs contain (or explain) a large percentage of the total variance and may be used to describe multivariate patterns or water quality variations across the watershed nearly as well as using the complete set of *p* original variables. These patterns are often related to specific sources of contamination [30].

The stepwise regression analysis was performed to determine the environmental or socio-economic factors (TP, P<0.10 and the others, P<0.05) that best explained the spatial variability of an individual river water quality variable [20]. Variables that were strongly intercorrelated with others (variance inflation factor >10) in the initial analysis, were removed and a further analysis was carried out with the remaining environmental variables for MRA and RDA [20], [31], [32]. Variance inflation factor (VIF) is a common way for detecting multicollinearity, a general rule is that the Variance Inflation Factor should not exceed 10 [49]. RDA was performed using Canoco 4.5 software to evaluate the relationships between river water quality and the environmental variables [33]. RDA was chosen because previous inspection of the data revealed a linear response rather than a unimodal response in the primary river water quality variables [34]. A Monte Carlo permutation test was used to verify the significance of the models [29]. Two series of matrices (i.e., for river water quality and environmental variables) from the RDA results were visualized as ordination diagrams using the CanoDraw software package for Windows. The river water quality parameters and the environmental/socio-economic variables were represented with arrows. Correlations between the watershed characteristics and/or the river water quality parameters were obtained by projecting the watershed characteristics onto each river water quality parameter in which higher values indicated higher correlations [34]. Application summary of 5 statistical methods in this study was listed in **Table S2**. The statistical analyses, except RDA, were completed using the SAS 9.1 software for Windows.

## Results

### 1 Physico-chemical water quality in the Cao-E River basin

**Figure 2:**
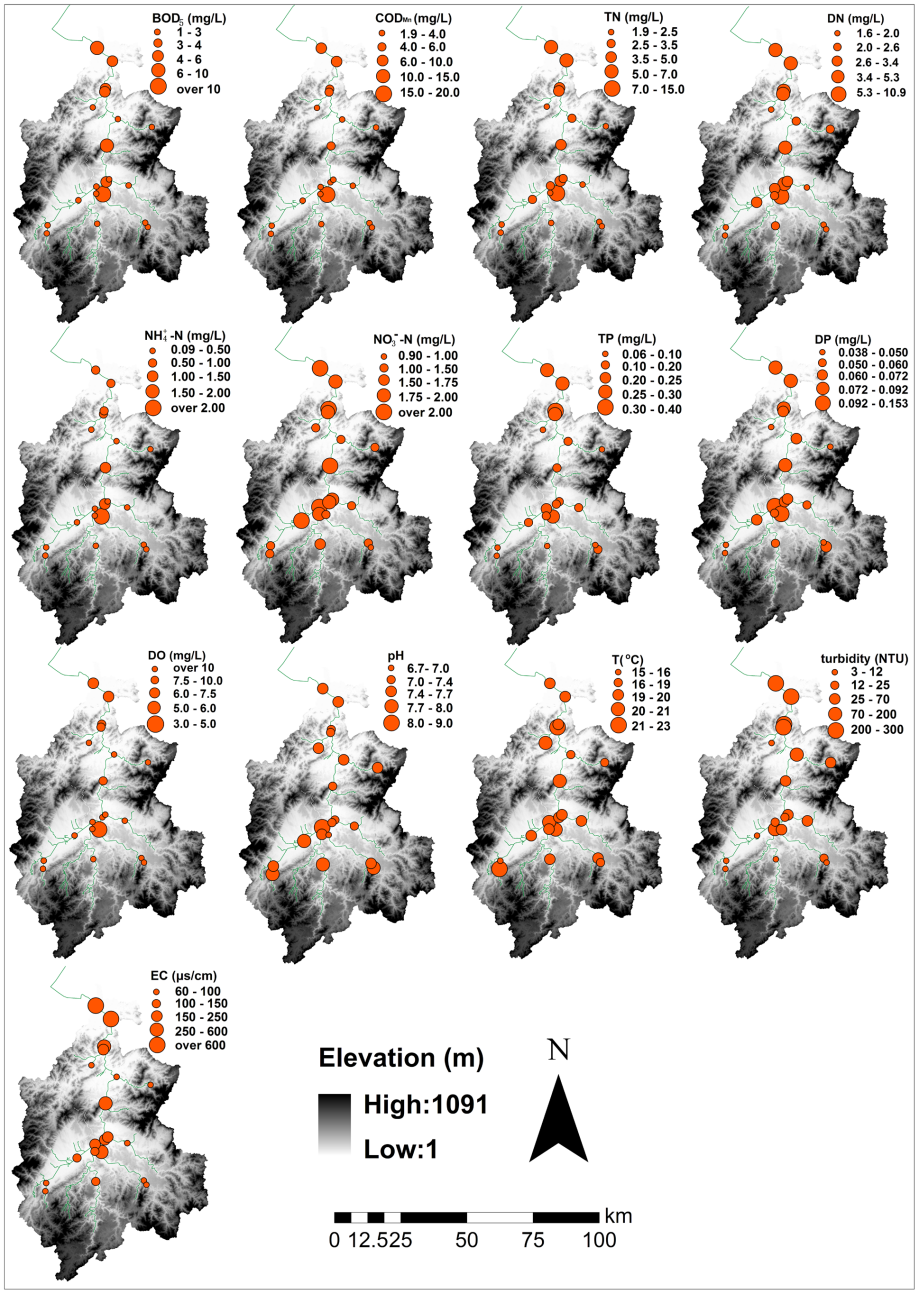
Spatial Variations In Bod5, Codmn, Tn, Dn, Nh_4_^+^-N, No_3_^−^-N, Tp, Dp, Do, Ph, T, Turbidity And Ec In The Study Area.

ANOVA was used to compare river water quality variations between the different seasons (i.e., rainy and dry). Temporal and spatial variations in the physico-chemical parameters are shown in **Table 2** and **Figure 2**. More specifically, BOD_5_, TN, DO, pH, T and turbidity exhibited significant temporal variations (**Table 2**, *p*<0.10). Moreover, TN, pH and T were generally higher in the rainy season (April to September), whereas higher values for BOD_5_, DO and turbidity occurred in the dry season (October to March). Most of the water quality parameters, except pH and T, exhibited considerable spatial variations. BOD_5_, COD_Mn_ and NH_4_^+^-N were higher in urban areas compared with surrounding rural areas, whereas DO exhibited a reverse response to the extent of urbanization. TN, DN, NO_3_^−^-N, TP and DP were higher in the lower part of the basin where more land has been developed and were lower in the upper-eastern part of the Cao-E River basin. Turbidity and EC were higher in the lower part of the basin (**Figure 2**) where the reach is often affected by tides and/or point source pollution [36], [37].

**Table 2:** Seasonal Averages, Coefficients Of Variation [Cv (%)] And River Water Quality Parameter Ranges.

Water Quality Parameters	Rainy Season		Dry Season		
	Average [Cv (%)]	Range	Average [Cv (%)]	Range	*P-Value*
Bod_5_ (Mg/L)	5.912 [183]	0.05–173.5	7.850 [181]	0–255.00	0.052
Cod_mn_ (Mg/L)	3.845 [87]	0.39–43.87	4.0361 [99]	0.49–47.75	0.557
Tn (Mg/L)	4.793 [103]	0.29–45.85	4.208 [73]	0.56–31.09	0.049
Dn (Mg/L)	3.549 [90]	0.06–22.53	3.295 [80]	0.03–30.07	0.219
Nh_4_^+^-N (Mg/L)	0.605 [195]	0–15.87	0.759 [2099]	0–19.58	0.156
No_3_^−^-N (Mg/L)	1.677 [41]	0.01–4.30	1.606 [69]	0.08–15.08	0.283
Tp (Mg/L)	0.150 [108]	0–1.88	0.168 [113]	0–3.86	0.317
Dp (Mg/L)	0.061 [91]	0–0.49	0.076 [253]	0–3.49	0.163
Do (Mg/L)	7.164 [33]	0–19.55	8.045 [42]	0–17.86	<0.001
Ph	7.538 [7]	5.33–9.34	7.449 [8]	5.45–10.12	0.055
T (°C)	24.967 [24]	8.40–37.20	14.262 [64]	4.70–105.00	<0.001
Turbidity (Ntu)	70.011 [105]	0.83–586.10	92.939 [113]	0.91–1111	0.002
Ec (µs/Cm)	306.38 [347]	14–14420	348.56 [282]	14–11230	0.557

The Probabilities Associated With The Student-Newman-Keuls Multiple-Range Test Are Also Provided.

Major gradients and principal patterns in the water quality data between the wet and dry seasons were detected using PCA. The PCA based on only the dry season water quality data indicated that 4 significant factors (i.e., PCs with an eigenvalue >1) explained 87.8% of the total variance (**Table 3**). The first factor accounted for 50.2% of the total variance and had a strong positive correlation with BOD_5_, COD_Mn_, TN, DN, NH_4_^+^-N, and T. The PCA of the wet season water quality data showed that the eigenvalues for the 2 most significant factors were 8.442 and 1.817, accounting for 78.9% of the total variance. Factor 1 accounted for 64.9% of the total variance and was positively correlated with BOD_5_, COD_Mn_, TN, DN, TP and DP (**Table 3**).

**Table 3:** River Water Quality Variables (13) On Rotated Pcs For Datasets During The Dry And Wet Seasons.

Water Quality Parameters	Dry Season				Wet Season	
	Pc1	Pc2	Pc3	Pc4	Pc1	Pc2
Bod_5_ (Mg/L)	0.354	−0.110	0.008	0.076	0.312	−0.041
Cod_mn_ (Mg/L)	0.350	0.200	−0.187	0.128	0.310	0.263
Tn (Mg/L)	0.380	0.001	0,077	0.083	0.333	−0.054
Dn (Mg/L)	0.362	−0.044	0.147	0.127	0.311	−0.197
Nh_4_^+^-N (Mg/L)	0.345	−0.215	0.019	0.084	0.267	−0.346
No_3_^−^-N (Mg/L)	0.275	−0.020	0.425	−0.079	0.283	0.174
Tp (Mg/L)	−0.221	0.409	0.077	−0.476	0.314	0.200
Dp (Mg/L)	0.097	0.329	0.609	−0.090	0.300	−0.018
Do (Mg/L)	−0.270	0.361	0.091	−0.204	0.272	0.349
Ph	−0.146	0.328	0.268	0.614	−0.184	0.546
T (°C)	0.418	−0.420	0.119	−0.212	0.010	0.246
Turbidity (Ntu)	0.222	0.340	−0.416	−0.312	0.283	0.246
Ec (µs/Cm)	0.241	0.308	−0.335	0.382	0.247	0.395
Eigenvalues	6.522	2.344	1.518	1.028	8.442	1.817
Percentage Of Variance	0.502	0.180	0.117	0.079	0.649	0.140
Cumulative Percentage Of Variance	0.502	0.682	0.799	0.878	0.649	0.789

Pcs Refer To Principal Components. The Significant Factors (I.E., Pcs With An Eigenvalue >1) Are List In The Table.

### 2 Relationships between watershed characteristics and river water quality

Relationships between the river water quality variables and their corresponding sub-watershed land use, topography and socio-economic factors were explored using a Pearson correlation test, MRA and RDA. All environmental variables, including land use, topography and socio-economic factors, were utilized to evaluate the response of river water quality to environmental gradients. The correlation analysis indicated that CRP was positively correlated with TN, DN, NO_3_^−^-N, TP and DP (*r*>0.45, *p*<0.05; *r* refers to the correlation coefficient). Moreover, UB was positively correlated with BOD_5_, COD_Mn_, TN, DN, NH_4_^+^-N, TP, DP and EC (*r*>0.50, *p*<0.05) and negatively correlated with DO and pH (*r*<−0.65, *p*<0.01). FRT was positively correlated with DO and pH (*r*>0.47, *p*<0.05) and exhibited a negative correlation with BOD, COD, TN, DN, NH_4_^+^-N, NO_3_^−^-N TP, and DP (*r*<−0.50, *p*<0.05). Furthermore, SLP_mean_ was positively correlated with DO (*r* = 0.59, *p*<0.01) and negatively correlated with COD_Mn_, TN, DN, NH_4_^+^-N, NO_3_^−^-N, TP, DP, T, turbidity and EC (*r*<−0.48, *p*<0.05). The elevation was positively correlated with DO (*r* = 0.45, *p*<0.05) and negatively correlated with COD_Mn_, TN, DN, NO_3_^−^-N, TP, DP, turbidity and EC (*r*<−0.47, *p*<0.05). Additionally, GDP_pc_ exhibited a positive correlation with COD, TN, DN, NO_3_^−^-N, TP, DP, turbidity, and EC (*r*>0.44, *p*<0.05). AHO_pc_ was positively correlated with TN, NO_3_^−^-N, TP, DP, turbidity and EC (*r*>0.47, *p*<0.05). Lastly, HP_d_ exhibited a negative correlation with DO and pH (*r*<−0.52, *p*<0.05) and a positive correlation to all other river water quality parameters (*r*>0.44, *p*<0.05) (**Table 4**).

**Table 4:** The Pearson Correlation Coefficients For The River Water Quality Parameters Compared With The Corresponding Sub-Watershed Characteristics.

	Frt	Wt	Ub	Crp	Elv	Slp_mean_	Ar	Hp_d_	Gdp_pc_	Aho_pc_
Bod_5_	−0.52*	0.11	0.76**	0.35	−0.31	−0.40	−0.08	0.59**	0.32	0.09
Cod_mn_	−0.61**	0.31	0.90**	0.40	−0.58	−0.64**	0.10	0.78**	0.60**	0.42
Tn	−0.72**	0.27	0.96**	0.51*	−0.61**	−0.74**	0.37	0.87**	0.60**	0.48*
Dn	−0.70**	0.17	0.91**	0.51*	−0.53*	−0.68**	0.34	0.81**	0.48*	0.40
Nh_4_^+^-N	−0.60**	0.15	0.84**	0.42	−0.39	−0.51*	0.14	0.69**	0.38	0.19
No_3_^−^-N	−0.54*	0.13	0.41	0.50*	−0.52*	−0.76**	0.70**	0.60**	0.45*	0.70**
Tp	−0.63**	0.37	0.77**	0.46*	−0.82**	−0.76**	0.46**	0.77**	0.66**	0.80**
Dp	−0.60**	0.08	0.61**	0.50*	−0.48*	−0.63**	0.30	0.64**	0.45*	0.48*
Do	0.48*	−0.43	−0.75**	−0.29	0.45*	0.59**	−0.63**	−0.71**	−0.44	−0.36
Ph	0.53*	0.01	−0.66**	−0.42	0.37	0.33	−0.44	−0.53*	−0.16	−0.15
T	−0.43	0.18	0.44	0.36	−0.44	−0.49*	0.46	0.53*	0.23	0.33
Turbidity	−0.28	0.40	0.42	0.15	−0.71**	−0.51*	0.36	0.45*	0.58**	0.81**
Ec	−0.21	0.61**	0.52*	0.02	−0.64**	−0.60**	0.20	0.57**	0.88**	0.78**

Abbreviations Are Provided In Table 1. The Figures In The Table Are Correlation Coefficients.

*Indicates Significance At The 0.05 Probability Level.

**Indicates Significance At The 0.01 Probability Level.

MRA demonstrated that no individual environmental factor was able to describe the overall water quality; however, most of the physico-chemical water parameters could be sufficiently predicted using 1 to 3 environmental factors (**Table 5**). Specifically, BOD_5_ could be predicted using UB, AR and GDP_pc_; COD_Mn_ with UB and AR; TN, DN, pH and DO with UB; NH_4_^+^-N with UB and HP_d_; DP with HP_d_; turbidity with AHO_pc_; EC with GDP_pc_; TP with UB and AHO_pc_; and NO_3_^−^-N with UB and SLP_mean_.

**Table 5:** Stepwise Multiple Regression Models For The River Water Quality Parameters And Watershed Characteristics In The Cao-E River Basin In Eastern China.

Parameters	Independent Variables	Regression Equation	R^2^	Adjusted R^2^		Significance
Bod_5_	Ub, Ar, Gdp_pc_	1.053+566.924ub-0.012ar-0.015gdp_pc_	0.856	0.830	**	
Cod_mn_	Ub, Ar	1.451+88.727ub-0.002ar	0.903	0.892	**	
Tn	Ub	1.668+69.168ub	0.912	0.907	**	
Dn	Ub	1.231+53.629ub	0.821	0.811	**	
Nh_4_^+^-N	Ub, Hp_d_	1.407+61.854ub-1.030hp_d_	0.805	0.782	**	
No_3_^−^-N	Slp_mean_, Ub	5.161012-0.233slp_mean_-7.675ub	0.720	0.670	**	
Tp	Ub, Aho_pc_	0.012+1.093ub+0.050aho_pc_	0.794	0.770	*	
Dp	Hp_d_	0.022+0.015hp_d_	0.406	0.373	**	
Do	Ub	9.802-50.534ub	0.562	0.538	**	
Ph	Ub	7.724012-5.814ub	0.439	0.408	**	
Turbidity	Aho_pc_	−115.264+96.209aho_pc_	0.662	0.643	**	
Ec	Gdp_pc_	−261.761+1.009gdp_pc_	0.778	0.766	**	

Abbreviations Are Provided In Table 1. The Parameters Without Regression Models Are Not Listed.

*Indicates Significance At The 0.05 Probability Level.

**Indicates Significance At The 0.01 Probability Level.

**Figure 3:**
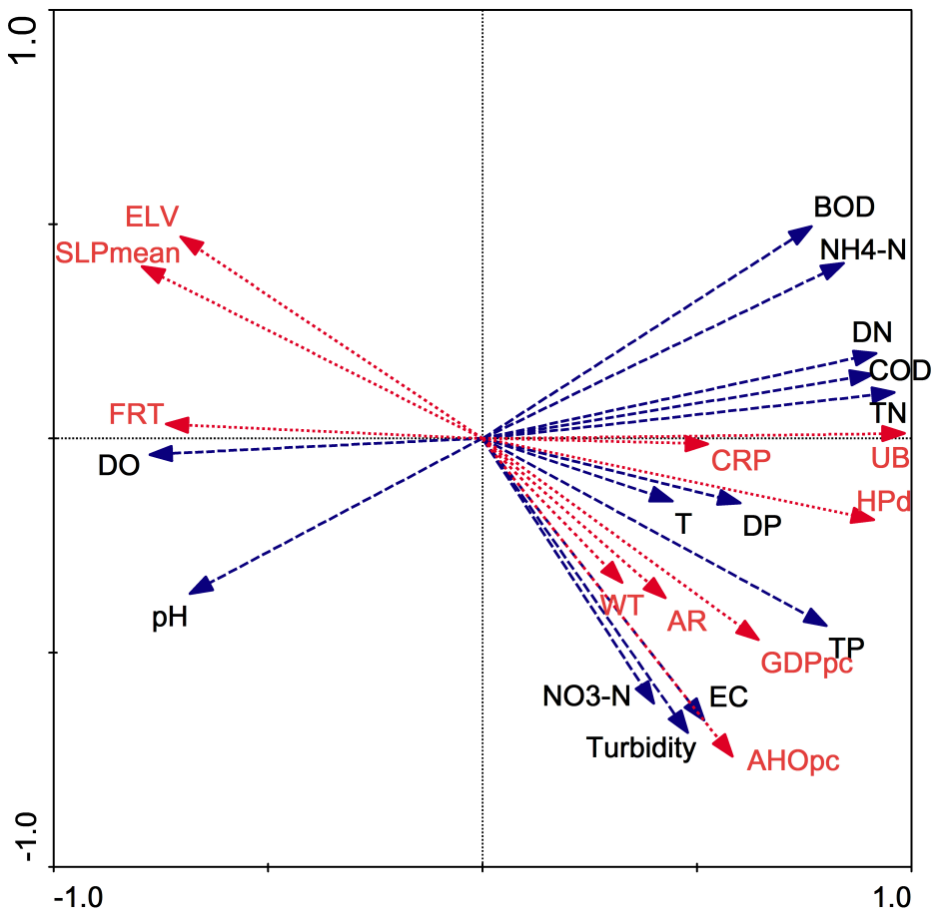
Biplots Of The River Water Quality Parameters And Watershed Characteristics In The Cao-E River Basin According To The Redundancy Analysis.

The RDA of the overall water quality as the dependent variable suggested that FRT, UB, CRP, ELV, SLP_mean_, AR, HP_d_, GDP_pc_ and AHO_pc_ explained 15.7–50.8% of the river water quality spatial variation, and a combination of the topography, land use and socio-economic factors explained 86.1% of the variation in overall water quality (**Table 6**). The ordination diagram of overall water quality and topography, land use and socio-economic factors indicated that the first RDA axis displayed a pollution gradient (e.g., COD_Mn_ and TN increased along the axis), which was positively correlated with UB and HP_d_ and negatively correlated with FRT and SLP_mean_ (**Figure 3**), accounting for 52.4% of the total variance in water quality (**Table 7**). The second axis was related to AHO_pc_ and only explained 16.5% of the total variance (**Table 7**).

**Table 6:** The Rda Results For The Percentage Of The Overall River Water Quality Variance Explained By An Individual Variable And All The Explanatory Variables.

Explanatory Variables	Variance Explained%	*P* Value
***Land Use***		
Frt	29.6	0.002
Wt	8.8	0.16
Ub	50.8	0.002
Crp	16.2	0.016
***Topography***		
Elv	29.8	0.004
Slp_mean_	36.3	0.002
Ar	15.7	0.032
***Socio-Economic Factors***		
Hp_d_	44.6	0.002
Gdp_pc_	26.2	0.032
Aho_pc_	26.9	0.002
Land Use + Topography + Social-Economic Factors	86.1	0.002

Abbreviations Are Provided In Table 1.

**Table 7:** The Rda Results Showing The Eigenvalues And Percentage Of The Overall River Water Quality Parameter Variance Explained By The First Two Rda Axes And All The Rda Axes.

	Axis 1	Axis 2	All Axes
Eigenvalues	0.524	0.165	0.861
Percentage Of Water Quality Variance Explained	52.4	16.5	86.1
Dominant Environmental Factors	Ub(0.967)	Aho_pc_(−0.699)	
	Hp_d_ (0.897)		
	Slp_mean_(−0.780)		
	Frt (−0.725)		

Abbreviations Are Provided In Table 1. The Number In Parentheses Indicates The Canonical Coefficients Of The Environmental Variables With The First Two Rda Axes.

## Discussion

### 1 Seasonal effects

Seasonal variations in the flow caused by the subtropical monsoon climate can partially explain the river water quality temporal variations [7], [38]. The relatively high TN concentrations in the rainy season were attributed to a flushing effect [36]. BOD_5_, which corresponds to point source pollution, were lower in the rainy season due to the dilution by precipitation [2]. Relatively high T in the rainy season resulted from the specific climatic conditions during this period (T is higher in the rainy season than in the dry season; see **Table 2**). The lower values of DO and higher values of pH in the rainy season may related to some environmental factors variations, such as flow rate, water temperature, aquatic plant growth and so on [50], [39]–[41]. The reasons are complex and the further research is needed in the future. The turbidity was higher in the dry season, which may be primarily because the reduced flow during this period caused the river water to be easily influenced by tides. Moreover, Odokuma and Okpokwasili [40] reported that the other parameters such as phosphate showed significantly higher values in the rainy season than in the dry season in the New Calabar River, Nigeria. These different results compared with our study were mainly due to the regional climate difference. However, the absence of a significant difference in the other physico-chemical parameters between the dry and wet seasons indicated mixed and irregular influences, e.g., point sources and diffusion may have played an important role [1], [42].

### 2 Land cover effects

In the study area, land-use types could play important roles in affecting major river water quality parameter distributions, as reflected by their considerable variability (**Figure 2**). This result is corroborated by the strong positive correlations between urban land and BOD_5_, COD_Mn_, TN, DN, NH_4_^+^-N and TP (**Table 5**) and the strong negative correlations with DO and pH. Moreover, most of the nutrient variables exhibited lower concentrations in the forest-dominated region, whereas the cropland-dominated region had high nutrient concentrations. The region downstream of the urban area exhibited higher concentrations of BOD_5_, COD, NH_4_^+^-N, TP and DP and lower pH and DO (**Figure 2**); this finding agrees with many related studies [21], [43], [44]. Studies have also shown that certain river water quality parameters (e.g., TN and TP) are primarily determined by agricultural land use at the watershed scale in many parts of the world [1], [20]. However, this study found that agricultural land use in watersheds plays a minor role in explaining the spatial variations in river water quality (explaining 16.2% of the total overall water quality variance) in the Cao-E River basin. The different result is primarily due to the enhanced effects of urban runoff and incompletely treated industrial waste and domestic pollution on river water quality compared to agricultural runoff. In addition, vegetated buffers adjacent to the croplands can substantially mitigate agricultural runoff of nutrients and other contaminants via deposition, absorption, and denitrification [20].

Urban areas are primarily located along river networks in the Cao-E River basin. Therefore, urban effects on the river water quality were expected. Urban land use comprised a much smaller percentage of the Cao-E River basin than the cropland in this study. However, as observed from the regression model (BOD_5_, COD_Mn_, TN, DN, NH_4_^+^-N, NO_3_^−^-N, TP, DO and pH) and the RDA analysis (explaining 50.8% of the total overall water quality variance) results, urban areas play a pivotal role in influencing water quality (**Tables 5** **and** **6**). This observation is most likely due to two factors: (1) the deficient capacity of urban sewage treatment plants leads to domestic pollution and industrial wastewater inputs into the Cao-E River system, and (2) the high percentage of impervious surfaces and over fertilization of grassy areas can increase discharge rates, sedimentation and pollutant runoff to streams [44].

### 3 Topography effects

Topographical factors played important roles in explaining spatial variations in river water quality within the Cao-E River basin (SLP_mean_ was 36.3% and ELV was 29.8%). Topography largely regulated the river water quality parameters. Most related studies have shown that higher SLP_mean_ and/or ELV lead to higher erosion rates, which subsequently increase the rate at which particulate matter enters a water body [13], [21], [45]. However, our results indicated an inverse relationship. Here, SLP_mean_ and ELV were both negatively correlated with TN, DN, NO_3_^−^-N, TP and DP; SLP_mean_ exhibited a highly significant negative correlation with NO_3_^−^-N values (**Figure 3** **and** **Table 5**). The slope effects on water chemistry varied. Watershed physical characteristics, such as soil properties (soil texture and soil drainage), morphological variables (drainage density and elongation) and particularly surficial debris, largely affected water chemistry in river waters [37], [46]. In addition, these negative correlations existed primarily because ELV and SLP_mean_ were strongly correlated with land cover (**Table 8**). High elevations and/or steep catchments are primarily occupied by forest land cover; however, cropland is primarily located in regions in which the topography is relatively flat and has a low ELV. Therefore, the forested mountain area, which has higher SLP_mean_ and/or ELV, may export fewer nutrients than flat land (e.g., cropland). Similar results were published regarding water quality spatial variations in the Han River basin in South Korea [7] and non-point source effects on stream nutrient concentrations in the Seattle region of the USA [47].

**Table 8:** The Pearson Correlation Matrix For The Watershed Characteristics Of The Cao-E River Basin In Eastern China.

	Frt	Wt	Ub	Crp	Elv	Slp_mean_	Ar	Hp_d_	Gdp_pc_	Aho_pc_
Frt	1.00									
Wt	0.09	1.00								
Ub	−0.79**	0.31	1.00							
Crp	−0.96**	−0.31	0.59**	1.00						
Elv	0.57**	−0.48*	−0.71**	−0.41	1.00					
Slp_mean_	0.83**	−0.33	−0.82**	−0.70**	0.74**	1.00				
Ar	−0.44	0.17	0.42	0.37	−0.44	−0.56*	1.00			
Hpd	−0.79**	0.37	0.95**	0.60**	−0.79**	−0.91**	0.55*	1.00		
Gdp_pc_	−0.38	0.49*	0.69**	0.18	−0.65**	−0.67**	0.30	0.76**	1.00	
Aho_pc_	−0.47*	0.20	0.55*	0.36	−0.69**	−0.72**	0.47*	0.64**	0.72**	1.00

*Indicates That The Correlation Was Significant At The 0.05 Probability Level.

**Indicates That The Correlation Was Significant At The 0.01 Probability Level.

### 4 The effects of socio-economic factors

The socio-economic factors commonly have different extent effects on river water quality, especially in developing countries such as China. Our study shows that socioeconomic factors have significant effects on river water quality during the study period (**Table 5**) and that HP_d_, AHO_pc_, and GDP_pc_ explain 44.6%, 26.9% and 26.2% of the total overall river water quality variance, respectively (**Table 6**). Furthermore, HP_d_ is a fundamental parameter for predicting DP and AHO_pc_ for TP and turbidity (**Table 5**). This result can be attributed to high population density, the massive animal production and the lack of strict management regulations for animal waste in the study area. The population density exceeds 380 people km^−2^, the annual animal output value exceeds 2.0 billion Yuan and the annual hog production exceeds 1.0 million heads for the entire basin [48]. Most human and animal waste with incomplete treatment is discharged into the surrounding water bodies. Moreover, EC was positively correlated with GDPpc, which was most likely related to point source pollution [37].

In addition, some researches [4]–[6] point out that climate condition is also an important factor of influencing river water quality. Thus, we will further study it in our future work.

## Conclusions

The primary results of this study can be summarized as follows:

The in-stream water quality in the Cao-E River basin streams suggested that TN, pH and T were generally higher in the rainy season, whereas BOD_5_, DO and turbidity were higher in the dry season.
Spatial variations in river water quality are typically associated with several anthropogenic and natural factors. Urban land cover was determined to be the most important explanatory variable for BOD_5_, COD_Mn_, TN, DN, NH_4_^+^-N, NO_3_^−^-N, TP, DO and pH. Moreover, Animal husbandry output per capita was an important predictor for TP and turbidity, and Gross domestic product per capita largely determined the spatial variations in EC. The remaining unexplained variance resulted from other factors, such as topography.
3) Pollution control for animal waste discharge in rural settlements was important in the study area. Moreover, agricultural runoff, industrial pollution and domestic pollution in urban and industrial areas were also important factors within the Cao-E River basin.
The percentage of total overall river water quality variance explained by individual variables and/or all environmental variables in the study area (as determined using RDA) can assist in quantitatively identifying primary pollution control factors at the watershed scale.

This study improved our understanding of the anthropogenic activities and natural factors that affect river water quality and can assist in the design of efficient strategies for controlling river water pollution at the watershed scale. Moderate-resolution remote sensing data were adopted in this study; future investigations will require high-resolution DEM maps and additional land use classes to better evaluate the effects of specific environmental variables on overall river water quality.

## Supporting Information

Table S1 Summary of commonly used statistical methods on pollution source identification in recent years.
(DOC)

Table S2 Application summary of 5 statistical methods in this study.
(DOC)
