# Supplementary material for: Effects of Land Use, Topography and Socio-Economic Factors on River Water Quality in a Mountainous Watershed with Intensive Agricultural Production in East China
Source: PLoS One. 2014 Aug 4;9(8):e102714. doi: 10.1371/journal.pone.0102714 (PMC4121078; doi:10.1371/journal.pone.0102714)
Supplement: Table S1 — Summary of commonly used statistical methods on pollution source identification in recent years. (DOC) [file pone.0102714.s001.doc]

**Supplementary data of Table S1**
