# Supplementary material for: Effects of Land Use, Topography and Socio-Economic Factors on River Water Quality in a Mountainous Watershed with Intensive Agricultural Production in East China
Source: PLoS One. 2014 Aug 4;9(8):e102714. doi: 10.1371/journal.pone.0102714 (PMC4121078; doi:10.1371/journal.pone.0102714)
Supplement: Table S2 — Application summary of 5 statistical methods in this study. (DOC) [file pone.0102714.s002.doc]

**Supplementary data of Table S2**

**Table S2** Application summary of 5 statistical methods in this study

| Methods used | Considered watershed characteristics | the outputs |
| --- | --- | --- |
| Analysis of variance (ANOVA) | no | whether there was a signiﬁcant diﬀerence during two seasons (*p* value). |
| Pearson correlation analysis | land use, topography and socio-economic factors | the Pearson correlation coefficient (r). |
| Multiple regression analysis  (MRA) | land use, topography and socio-economic factors | multiple regression equations |
| Principal component analysis (PCA) | no | river water quality variables (13) on rotated PCs for datasets during two seasons. |
| Redundancy analysis (RDA) | land use, topography and socio-economic factors | 1) the percentage of the overall water quality variance explained by an individual (or all) explanatory variables.  2) the percentage of the overall river water quality parameter variance explained by the RDA axes.  3) biplots of the river water quality parameters and watershed characteristics. |

Water quality parameters are considered including BOD_5_, COD_Mn_, TN, DN, NH_4_^+^-N, NO_3_^－^-N, TP, DP, DO, pH, Turbidity and EC.

Watershed characteristics includes Land use (FRT, WT, UB and CRP), Topography (ELV, SLP_mean_ and AR) and Socio-economic factors (HP_d_, GDP_pc_ and AHO_pc_ )
